# Safety and Efficacy of Stereotactic Body Radiation Therapy in Very Elderly Patients (≥80 Years) with Solitary Hepatocellular Carcinoma

**DOI:** 10.3390/cancers18111809

**Published:** 2026-06-01

**Authors:** Yuki Tamura, Hiroki Tojima, Daichi Takizawa, Mitsuhiko Shibasaki, Hirotaka Arai, Hiroki Kiyohara, Yukihiko Yoshimatsu, Takashi Ueno, Takashi Kosone, Toru Fukuchi, Takayoshi Suga, Shuichi Saito, Hideyuki Suzuki, Yuichi Yamazaki, Satoru Kakizaki, Toshio Uraoka

**Affiliations:** 1 Department of Gastroenterology and Hepatology, Gunma University Graduate School of Medicine, Maebashi 370-0829, Japan; y_tamura@gunma-u.ac.jp (Y.T.);; 2 Department of Gastroenterology, Japanese Red Cross Maebashi Hospital, Maebashi 371-0811, Japan; 3 Department of Radiation Oncology, Japanese Red Cross Maebashi Hospital, Maebashi 371-0811, Japan; 4 Department of Internal Medicine, Isesaki Municipal Hospital, Isesaki 372-0817, Japan; 5 Department of Gastroenterology and Hepatology, Kusunoki Hospital, Fujioka 375-0024, Japan; 6 Department of Internal Medicine, Kiryu Kosei General Hospital, Kiryu 376-0024, Japan; 7 Department of Gastroenterology, NHO Shibukawa Medical Center, Shibukawa 377-0024, Japan; 8 Department of Gastroenterology, Tomioka Public General Hospital, Tomioka 370-2316, Japan; 9 Department of Internal Medicine, Japanese Red Cross Haramachi Hospital, Higashiagatsuma 377-0801, Japan; 10 Department of Clinical Research, NHO Takasaki General Medical Center, Takasaki 370-0829, Japan

**Keywords:** alternative treatment, clinical, elderly patients, hepatocellular carcinoma, radiation therapy

## Abstract

Background: The number of elderly patients with hepatocellular carcinoma (HCC) has been increasing worldwide. This study aimed to evaluate the clinical outcomes of stereotactic body radiation therapy (SBRT) for patients aged ≥80 years with solitary HCC. Methods: This retrospective study included 117 patients with solitary HCC treated with SBRT between January 2019 and June 2025 at eight institutions in Japan. Patients were categorized into the very elderly group (≥80 years, *n* = 41) and the control group (<80 years, *n* = 76). Overall survival (OS), local tumor progression (LTP), and treatment-related toxicities were evaluated. Propensity score matching (PSM) was performed to minimize baseline imbalances between the two groups. Results: The median age was 84 years in the very elderly group and 74 years in the control group. The 3-year OS rates were 79.0% in the very elderly group and 62.8% in the control group, with no statistically significant difference observed (crude cohort: 95% CI, 0.18–1.08, *p* = 0.072; PSM cohort: 95% CI, 0.12–1.26, *p* = 0.114). The 3-year LTP rates were 18.9% in the very elderly group and 15.6% in the control group, without significant intergroup differences. Acute toxicities were numerically more frequent in the very elderly group; however, no grade ≥3 toxicities or treatment-related deaths were observed. Changes in ALBI score did not differ significantly between groups (*p* = 0.785). Conclusions: SBRT may represent a feasible treatment option with an acceptable safety profile in carefully selected very elderly patients with solitary HCC. It may be a reasonable option for this growing patient population.

## Simple Summary

The number of very elderly patients with liver cancer is increasing worldwide, creating a need for safe and less invasive treatment options. Standard treatments such as surgery or local ablation may not be suitable for older patients because of physical burden or tumor location. Stereotactic body radiation therapy (SBRT) is a noninvasive treatment that precisely delivers high-dose radiation to tumors while sparing surrounding tissues. In this multicenter study, we compared outcomes between patients aged 80 years or older and younger patients treated with SBRT. We found that survival and tumor control were similar between the two groups. Although side effects were slightly more frequent in very elderly patients, they were generally mild and manageable. These findings suggest that SBRT may be a reasonable treatment option for carefully selected very elderly patients with liver cancer.

## 1. Introduction

As life expectancy increases worldwide, the number of individuals aged ≥80 years is expected to triple between 2020 and 2050, and the incidence of cancer among older adults is projected to increase [1,2]. In Japan, the age at diagnosis of hepatocellular carcinoma (HCC) has steadily increased [3].

Current guidelines recommend surgical resection, liver transplantation, or local ablation as first-line treatment options for localized HCC [4,5,6,7]. However, these treatments may be difficult to apply in elderly patients because of invasiveness, comorbidities, or impaired liver function. Percutaneous ablation therapies, including radiofrequency ablation (RFA) and microwave ablation (MWA), are less invasive and can be safely performed even in patients aged ≥80 years with early-stage HCC [8,9,10], although they may be technically challenging because of anatomical constraints. Several studies of local treatment for HCC have used 80 years as a threshold for older patients [9,11]. Stereotactic body radiation therapy (SBRT) has emerged as an alternative treatment for patients unsuitable for surgery or ablation [12,13]. Previous studies have shown favorable local control with SBRT; however, evidence in elderly patients remains limited [14,15,16]. This study aimed to evaluate the safety and efficacy of SBRT in patients aged ≥80 years with early-stage HCC.

## 2. Materials and Methods

This retrospective cohort study included patients with solitary intrahepatic HCC who were referred from eight institutions in Gunma, Japan, and subsequently treated with SBRT at Japanese Red Cross Maebashi Hospital between January 2019 and June 2025. HCC was diagnosed either pathologically or based on typical findings on contrast-enhanced dynamic CT and gadolinium ethoxybenzyl diethylenetriamine pentaacetic acid (Gd-EOB-DTPA)-enhanced MRI [4,5].

**Figure 1:**
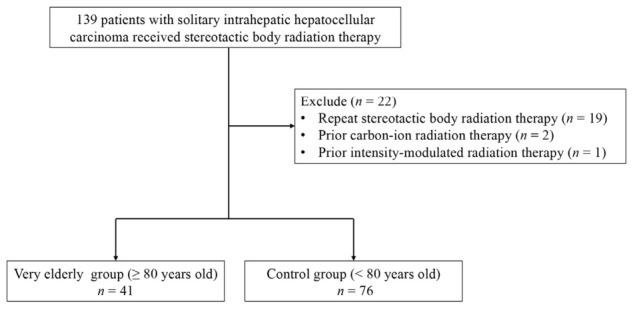
Patient selection flowchart.

Eligibility criteria were (i) primary or recurrent/residual solitary HCC; (ii) no prior SBRT or other radiation therapy, including carbon-ion radiation therapy (CIRT), to the target lesion; (iii) no repeat SBRT to the target or other hepatic lesion(s); (iv) no extrahepatic metastasis; (v) Eastern Cooperative Oncology Group (ECOG) performance status 0 or 1; and (vi) Child–Pugh class A or B. Nineteen patients were excluded because of repeat SBRT, two because of prior CIRT, and one because of prior intensity-modulated radiation therapy. Ultimately, 117 patients were included and categorized as the very elderly group (≥80 years; 41 patients) and the control group (<80 years; 76 patients) (Figure 1).

Most patients were considered unsuitable for RFA or surgical resection because of anatomical constraints, tumor size, treatment-related invasiveness, or proximity of the lesion to critical structures such as the inferior vena cava, portal vein, or small intestine. These patients were referred to Japanese Red Cross Maebashi Hospital, where their cases were re-evaluated at a multidisciplinary conference involving hepatologists and radiation oncologists. The decision to perform transcatheter arterial chemoembolization (TACE) before SBRT, hereafter referred to as “prior TACE,” was made individually based on patient and tumor characteristics. For example, patients with a history of TACE, tumors with weak contrast enhancement, and chronic kidney disease were considered less suitable for prior TACE. Furthermore, some patients preferred to undergo SBRT alone.

**Figure 2:**
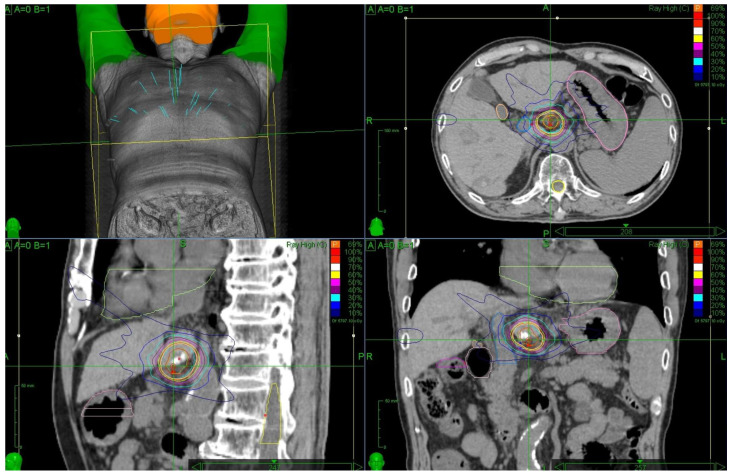
Axial, sagittal, and coronal views of a representative CyberKnife treatment plan with dose distribution. Isodose lines are overlaid to illustrate the dose distribution.

For SBRT, patients were immobilized with customized vacuum cushions and underwent contrast-enhanced four-dimensional CT simulation. Tumor delineation was performed using dynamic CT and MRI findings. Respiratory motion was incorporated by generating an internal target volume (ITV), followed by sequential 3-mm isotropic expansions to define the clinical and planning target volumes (Figure 2).

All SBRT planning, target delineation, dose prescription, and treatment delivery were performed at Japanese Red Cross Maebashi Hospital. The prescribed dose was up to 40 Gy in five fractions, based on previously reported SBRT protocols for HCC [17,18]. Dose constraints for organs at risk were defined as follows: for the spared liver, V_20Gy_ < 20%; for the stomach, V_26.5Gy_ < 5 cm^3^; and for the duodenum, V_18.5Gy_ < 5 cm^3^ and V_14.5Gy_ < 10 cm^3^ (Table 1). Vx was defined as the volume of an organ receiving at least x Gy. The dose was reduced to 35 Gy or 30 Gy in five fractions based on tumor location and organ-at-risk dose constraints. All treatments were delivered using the CyberKnife M6 system (Accuray Inc., Sunnyvale, CA, USA).

After completion of SBRT, patients underwent laboratory testing, including α-fetoprotein (AFP) and des-γ-carboxy prothrombin (DCP), every 1–3 months. Dynamic CT or EOB-MRI was performed approximately every 3–6 months to assess tumor response and detect recurrence, as evaluated by hepatologists and/or radiologists. Acute toxicities were defined as adverse events within 3 months after SBRT and graded according to the Common Terminology Criteria for Adverse Events, version 5.0. When recurrence was detected, subsequent treatment, including TACE, RFA, molecular targeted therapy, or immunotherapy, was selected at the discretion of the treating institution. LTP was defined as the appearance of viable tumor adjacent to the treated lesion on dynamic CT or MRI according to modified RECIST criteria [19]. Distant recurrence was defined as the development of new HCC nodules separate from the treated site. Overall survival (OS) was defined as the time from SBRT initiation to death from any cause.

All statistical analyses were performed using EZR version 1.68 (Saitama Medical Center, Jichi Medical University, Saitama, Japan) [20]. Categorical and continuous variables are presented as numbers (percentages) and medians with interquartile ranges (IQRs), respectively. Between-group comparisons were conducted using the chi-square test, Fisher’s exact test, or the Mann–Whitney U test, as appropriate. Kaplan–Meier curves were generated for survival outcomes and compared using the log-rank test. Hazard ratios (HRs), corresponding 95% confidence intervals (CIs), and *p*-values were estimated using Cox proportional hazards regression models. Multivariate analysis was performed using the Cox proportional hazards model to identify prognostic factors associated with OS. Clinically relevant variables or those with potential univariate associations were included. Within each group, albumin–bilirubin (ALBI) scores at 6 months after treatment were compared with baseline values using the Wilcoxon signed-rank test. Intergroup differences in longitudinal ALBI score changes were evaluated using a mixed-effects model, which accounted for repeated measurements within individuals and allowed inclusion of patients with incomplete follow-up data. ALBI score measurements obtained after additional locoregional or systemic therapies were excluded. Propensity score matching (PSM) was performed to reduce potential confounding and selection bias by balancing baseline characteristics between the groups. Matching variables included sex, ECOG performance status, viral etiology, tumor diameter, ALT, AFP, DCP, baseline ALBI score, and prescribed SBRT dose. One-to-one nearest-neighbor matching without replacement was conducted using a caliper width of 0.2 times the standard deviation of the logit of the propensity score. Death from any cause was treated as a competing event in analyses of LTP and distant recurrence. Cumulative incidence functions were estimated, and differences between groups were compared using Gray’s test. Additionally, the Fine–Gray subdistribution hazard model was used to estimate hazard ratios and corresponding 95% CIs. All tests were two-sided, and *p* < 0.05 was considered statistically significant.

## 3. Results

### 3.1. Patient Characteristics

Patient characteristics are summarized in Table 2. The median age was 84 years (81.0–86.0 years) in the very elderly group and 74 years (69.8–76.0 years) in the control group. There were no significant differences in comorbidities, including hypertension, hyperlipidemia, and diabetes mellitus, between the groups. In both groups, approximately half of the target lesions (50.4%) had no prior treatment, such as RFA or TACE, and were defined as treatment-naïve HCC. In contrast, most patients (72.6%) had a history of prior HCC and were therefore classified as having recurrent HCC. Compared with the control group, the very elderly group included a larger proportion of patients with ECOG performance status 1 (*p* = 0.040) and had lower ALT levels (*p* = 0.003). Radiation doses were reduced in approximately half of the patients in the very elderly group, but treatment protocols did not differ significantly between groups. After propensity score matching, no statistically significant differences in baseline characteristics were observed based on *p*-values (Table 3).

### 3.2. Short-Term Treatment Outcomes and OS in the Very Elderly and the Control Group

The median follow-up period was 25.5 months (95% CI, 13.8–39.6) in the very elderly group and 26.3 months (95% CI, 16.2–40.5) in the control group. During follow-up, 6 patients (14.6%) in the very elderly group and 27 (35.5%) in the control group died. In the very elderly group, deaths were attributed to HCC progression (*n* = 3), liver failure (*n* = 1), and other causes (*n* = 2).

**Figure 3:**
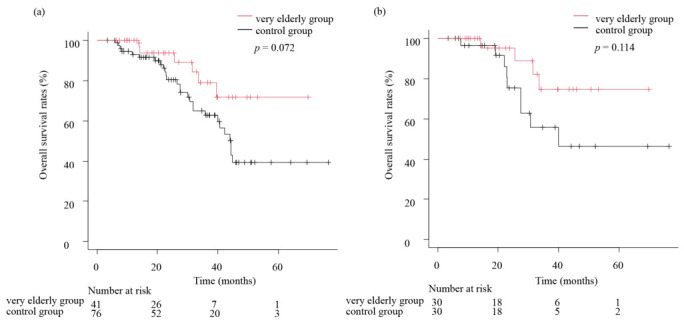
(**a**) Overall survival in the very elderly and control groups in the crude cohort. No significant difference was observed between the two groups (HR 0.44; 95% CI 0.18–1.08; *p* = 0.072), as the confidence interval crossed 1.0. (**b**) Overall survival in the very elderly and control groups in the matched cohort. No significant difference was observed between the two groups (HR, 0.39; 95% CI, 0.12–1.26; *p* = 0.114). SBRT, stereotactic body radiation therapy; CI, confidence interval; HR, hazard ratio.

In the control group, deaths were attributed to liver failure (*n* = 10), HCC progression (*n* = 9), and other causes (*n* = 8). No deaths were considered directly attributable to SBRT. In the crude cohort, median OS was not reached in the very elderly group and 44.2 months in the control group; the 1-, 2-, and 3-year OS rates were 100% (95% CI NA–NA), 93.6% (95% CI 76.9–98.4), and 79.0% (95% CI 55.7–90.9) in the very elderly group, and 93.1% (95% CI 84.1–97.1), 80.4% (95% CI 67.8–88.5), and 62.8% (95% CI 47.9–74.5) in the control group, respectively. No significant difference was observed between the two groups (HR 0.44; 95% CI 0.18–1.08, *p* = 0.072; Figure 3a). Univariate and multivariate Cox regression analyses were performed to identify factors associated with OS (Table 4). In multivariate analysis, age ≥ 80 years was not significantly associated with OS, whereas baseline ALBI score and male sex were independent prognostic factors.

After propensity score matching, median OS was not reached in the very elderly group and was 40.0 months in the control group. The 1-, 2-, and 3-year OS rates were 100% (95% CI NA–NA), 95.2% (95% CI 70.7–99.3), and 74.6% (95% CI 44.9–89.8) in the very elderly group, and 96.3% (95% CI 76.5–99.5), 75.3% (95% CI 50.1–89.1), and 55.8% (95% CI 29.8–75.5) in the control group, respectively. No significant difference was observed between the two groups (HR 0.39; 95% CI, 0.12–1.26; *p* = 0.114; Figure 3b).

### 3.3. LTP in the Very Elderly and the Control Groups

**Figure 4:**
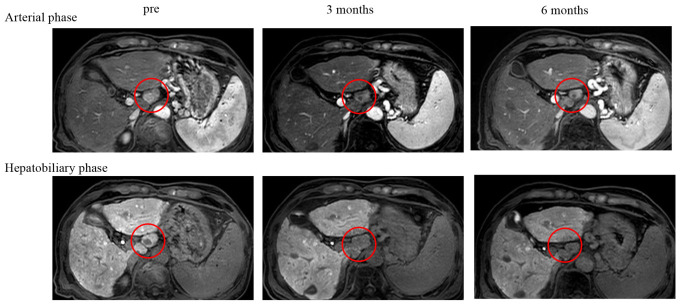
Representative gadolinium ethoxybenzyl diethylenetriamine pentaacetic acid-enhanced MRI demonstrating complete response. Arterial and hepatobiliary phase images before and at 3 and 6 months after SBRT are shown. At 3 months after SBRT, disappearance of arterial phase enhancement is observed (circle), consistent with complete response according to modified RECIST criteria.

**Figure 5:**
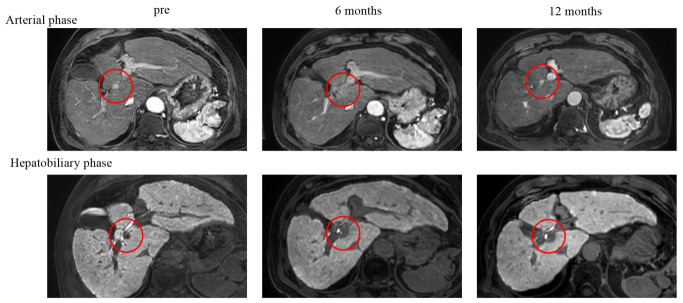
Representative gadolinium ethoxybenzyl diethylenetriamine pentaacetic acid-enhanced MRI demonstrating local tumor progression. Arterial and hepatobiliary phase images before and at 6 and 12 months after SBRT are shown. At 12 months, a newly appearing arterial phase enhancement adjacent to the treated lesion is observed (circle), consistent with local tumor progression.

LTP occurred in 3 patients (7.3%) in the very elderly group and 8 patients (10.5%) in the control group. Representative imaging findings demonstrating complete response and local tumor progression are shown in Figure 4 and Figure 5, respectively (Figure 4 and Figure 5).

**Figure 6:**
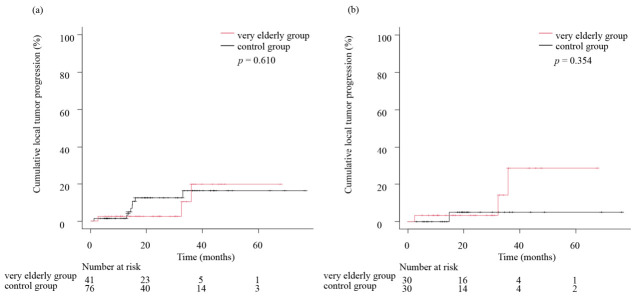
(**a**) Local tumor progression in the very elderly and control groups in the crude cohort. No significant difference was observed between the two groups (HR 0.71; 95% CI 0.19–2.67; *p* = 0.610). (**b**) Local tumor progression in the very elderly and control groups in the matched cohort. No significant difference was observed between the two groups (HR 2.92; 95% CI 0.30–28.0; *p* = 0.354). SBRT, stereotactic body radiation therapy; CI, confidence interval; HR, hazard ratio.

In the crude cohort, the 1-, 2-, and 3-year LTP rates were 2.4% (95% CI, 0.3–16.1), 2.4% (95% CI, 0.3–16.1), and 18.9% (95% CI, 5.8–52.2), respectively, in the very elderly group, and 1.3% (95% CI, 0.2–9.0), 11.9% (95% CI, 5.9–23.5), and 15.6% (95% CI, 7.7–30.1), in the control group, respectively. No significant difference was observed between the two groups. (HR 0.71; 95% CI, 0.19–2.67; *p* = 0.610; Figure 6a).

After matching, the 1-, 2-, and 3-year LTP rates were 3.3% (95% CI, 0.5–21.4), 3.3% (95% CI, 0.5–21.4), and 28.4% (95% CI, 8.8–70.3), respectively, in the very elderly group, and 0% (95% CI, NA–NA), 5.0% (95% CI, 0.7–30.5), and 5.0% (95% CI, 0.7–30.5), respectively, in the control group. No significant difference was observed (HR 2.92; 95% CI, 0.30–28.0; *p* = 0.354; Figure 6b). Additional competing risk analyses yielded similar findings, with no significant differences in LTP between the groups (HR 0.75; 95% CI 0.20–2.82; *p* = 0.670; Supplemental Figure S1).

### 3.4. Distant Recurrence in the Very Elderly Group and the Control Group

Distant recurrence was observed in 24 patients (58.5%) in the very elderly group and 49 patients (64.5%) in the control group.

**Figure 7:**
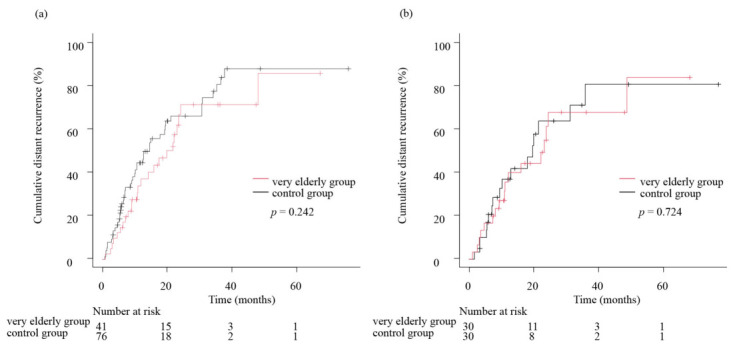
(**a**) Kaplan–Meier curves for distant recurrence in the crude cohort. No statistically significant difference was observed between the very elderly and control groups (HR 0.75; 95% CI 0.46–1.22; *p* = 0.242). (**b**) Kaplan–Meier curves for distant recurrence in the matched cohort, likewise, demonstrating no significant intergroup difference (HR 0.89; 95% CI 0.45–1.74; *p* = 0.724). SBRT, stereotactic body radiation therapy; CI, confidence interval; HR, hazard ratio.

In the crude cohort, the 1-, 2-, and 3-year distant recurrence rates were 33.9% (95% CI 21.1–51.4), 66.6% (95% CI 49.3–83.0), and 71.4% (95% CI 53.7–86.9) in the very elderly group and 44.4% (95% CI 33.6–57.0), 66.1% (95% CI 53.7–78.1), and 80.6% (95% CI 67.5–90.9) in the control group, respectively. No statistically significant difference in distant recurrence was identified between the groups (HR 0.75; 95% CI 0.46–1.22; *p* = 0.242; Figure 7a).

After propensity score matching, the corresponding distant recurrence rates at 1, 2, and 3 years were 35.7% (95% CI 20.8–56.6), 61.4% (95% CI 41.5–81.5), and 67.8% (95% CI 47.2–86.7) in the very elderly group and 37.0% (95% CI 21.7–58.2), 63.7% (95% CI 43.7–83.3), and 80.7% (95% CI 57.3–95.8) in the control group, respectively. Consistent with the crude cohort analysis, no significant intergroup difference was observed (HR 0.89; 95% CI 0.45–1.74; *p* = 0.724; Figure 7b). Competing-risk analysis also demonstrated similar findings (HR 0.79; 95% CI 0.50–1.27; *p* = 0.340; Supplemental Figure S2).

### 3.5. Acute Toxicities

In the very elderly group, 12 patients (29.3%) experienced acute toxicities, most commonly fatigue (*n* = 5) and abdominal discomfort (*n* = 3), followed by diarrhea (*n* = 2), esophagitis (*n* = 1), and bile duct stenosis (*n* = 1). In the control group, 11 patients (14.5%) experienced acute toxicities, including fatigue (*n* = 5), abdominal discomfort (*n* = 3), γ-glutamyl transferase elevation (*n* = 1), radiation dermatitis (*n* = 1), and ascites (*n* = 1). No radiation-induced liver disease (RILD) was observed three months or more after SBRT. Although acute toxicities were numerically more frequent in the very elderly group, no significant difference was detected (*p* = 0.086). All toxicities resolved with conservative management or medication. No grade ≥ 3 adverse events occurred in either group.

### 3.6. Changes in ALBI Score

**Figure 8:**
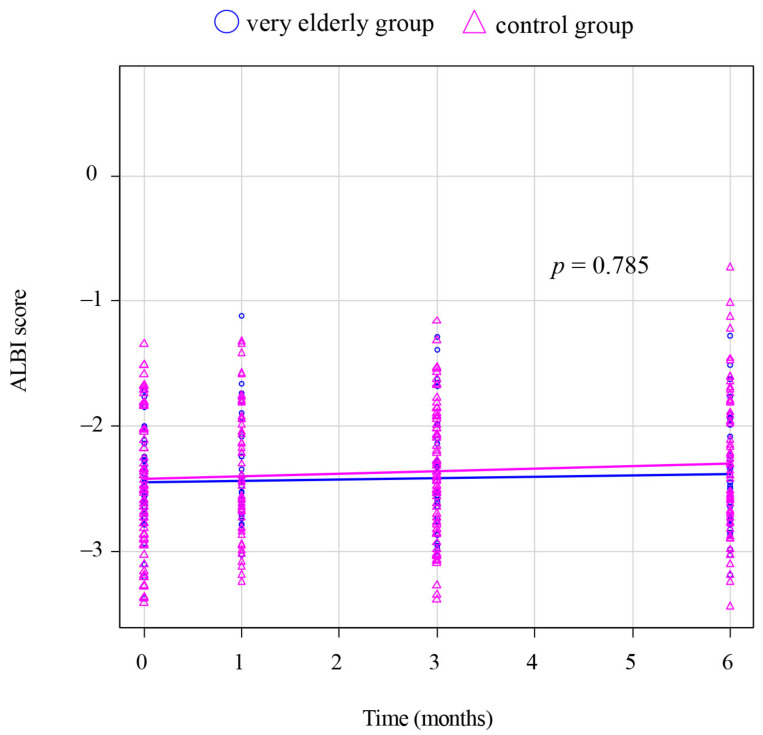
Scatter plots showing changes in ALBI scores from baseline to 6 months after each treatment. Lines indicate the mean values. Blue and red lines represent the very elderly and the control groups, respectively. The mean ALBI scores in the very elderly and control groups were −2.50 ± 0.36 and −2.48 ± 0.52 at baseline, and −2.40 ± 0.44 and −2.29 ± 0.59 at 6 months, respectively. The between-group difference in ALBI score change was not statistically significant (*p* = 0.785). ALBI, albumin–bilirubin; SBRT, stereotactic body radiation therapy.

To assess changes in liver function, ALBI scores at baseline, 1, 3, and 6 months after treatment were evaluated. In the very elderly group, the mean ALBI score changed from −2.50 ± 0.36 at baseline to −2.40 ± 0.44 after treatment (*p* = 0.058). In the control group, the mean ALBI score changed from −2.48 ± 0.52 to −2.29 ± 0.59 (*p* < 0.001). Although a significant increase in ALBI score was observed in the control group, the between-group difference in ALBI score change was not statistically significant (*p* = 0.785; Figure 8; Supplemental Figure S3).

## 4. Discussion

The main findings of the present study suggest that SBRT may represent a feasible and effective treatment option for carefully selected patients aged ≥80 years with early-stage HCC. No significant differences were observed in OS or LTP between the very elderly and control groups. Although acute toxicities were numerically more frequent in the very elderly group, all events were manageable and did not lead to treatment-related death. Although ALBI scores tended to worsen after SBRT, the magnitude of change did not differ significantly between groups. Previous studies have demonstrated favorable disease control with SBRT, but evidence regarding its safety and efficacy in very elderly patients remains limited [14,15]. To our knowledge, this is one of the first comparative studies specifically evaluating SBRT in patients aged ≥80 years with early-stage HCC.

The number of individuals aged ≥80 years is projected to triple between 2020 and 2050 [1]. In Japan, the age at diagnosis of primary HCC has steadily increased, and several studies have reported the efficacy and safety of RFA or MWA in patients aged ≥80 years with early-stage HCC [2,9,10]. Although Teraoka et al. compared patients aged ≥75 years with younger patients and reported comparable OS with acceptable safety profiles, no comparative study has specifically evaluated SBRT in very elderly patients aged ≥80 years [21]. Given the increasing proportion of very elderly patients in clinical practice, establishing evidence supporting SBRT as an alternative treatment in this population is of growing importance.

In the present study, the 3-year OS rate in patients aged ≥80 years was approximately 79%. Although no statistically significant difference was detected between groups, the control group exhibited somewhat lower OS (crude cohort, HR 0.443, *p* = 0.072; PSM cohort, HR 0.386, *p* = 0.114). Loi et al. reported a 2-year OS rate of 43% in patients aged ≥80 years treated with SBRT [11], whereas our elderly cohort demonstrated more favorable outcomes. In addition to possible selection bias, this discrepancy may reflect differences in baseline tumor burden and clinical characteristics between the studies. In the study by Loi et al., 60% of patients had BCLC stage B disease and 78% had received prior local treatment, suggesting a population with more advanced or recurrent disease. In contrast, published studies including patients with recurrent or residual HCC have reported 2- to 3-year OS rates of approximately 66–69%, which are comparable to those observed in our control group [22,23]. In the present study, the very elderly group had significantly lower ALT levels (*p* = 0.003) and numerically lower proportions of Child–Pugh score ≥ 7 (7.3% vs. 14.5%) in the crude cohort. These baseline differences may indicate that very elderly patients with relatively preserved liver function and favorable tumor characteristics were more likely to be selected for SBRT. Therefore, the numerically higher OS observed in the very elderly group should be interpreted cautiously, as it may reflect selection bias and a potential healthy survivor effect rather than a true survival advantage. Nevertheless, these findings suggest that SBRT may be considered a treatment option for selected very elderly patients.

The 3-year LTP rates were 18.9% in the very elderly group and 15.6% in the control group. Although these rates were somewhat higher than those reported in studies of previously untreated solitary HCC, no significant difference in LTP was observed between groups (crude cohort, *p* = 0.610; PSM cohort, *p* = 0.354) [24]. These findings should be interpreted in the context of our study population, in which a substantial proportion of patients had recurrent disease (72.6%) and nearly half of the target lesions had been previously treated (49.6%). Shui et al. reported a 3-year freedom from local progression rate of 82% in patients with recurrent HCC, while Kibe et al. reported 3-year cumulative local progression rates ranging from 5.0% to 14.8% in patients with recurrent or residual HCC, which are consistent with our results [23,25]. Although local control in recurrent or residual HCC may be more challenging than in previously untreated HCC, our findings suggest that SBRT appeared to provide comparable local control in very elderly patients.

SBRT appeared to be generally well tolerated in very elderly patients, with toxicity profiles and liver function outcomes similar to those in younger patients. Previously, the use of radiation therapy for liver tumors was limited because of the risk of RILD associated with irradiation of large volumes of non-tumorous hepatic parenchyma at high radiation doses [26]. In contrast, SBRT delivers ablative radiation doses to target lesions with tight treatment margins and limited fractionation, thereby minimizing exposure of normal liver tissue and reducing the risk of RILD [27]. A meta-analysis by Sawrie et al. of studies involving SBRT for HCC and liver metastases reported an RILD incidence of 2.4% [28]. Teraoka et al. reported no significant difference in adverse events between patients aged ≥75 years and younger patients, whereas Loi et al. observed a relatively higher incidence of acute toxicities of 28% in patients aged ≥80 years, which is comparable to the rate observed in the present study [11,21]. In our study, acute toxicities were numerically more frequent in the very elderly group (29.3%) than in the control group (14.5%); however, no statistically significant difference was observed (*p* = 0.086). This finding may reflect limited statistical power due to the relatively small sample size rather than a true absence of differences in treatment tolerability between groups. Although no grade ≥ 3 toxicities were observed and all adverse events resolved with conservative or appropriate medical management, the possibility that acute toxicities may occur more frequently in very elderly patients should be recognized. Taken together, these findings suggest that acute toxicities after SBRT may be somewhat more frequent in very elderly patients, but are generally manageable.

Liver function was evaluated based on changes in ALBI score from baseline to 6 months after SBRT. Several studies have reported significant deterioration in ALBI scores following SBRT [29,30]. In the present study, ALBI scores increased modestly in both groups. Although statistically significant within-group deterioration was observed only in the control group (*p* < 0.001), the magnitude of longitudinal ALBI score change did not differ significantly between the groups (*p* = 0.785). The significantly higher ALT levels in the control group may have contributed to the greater deterioration in liver function observed after SBRT. In addition, previous studies have reported that patients with baseline modified ALBI (mALBI) grade 2a and 2b are more susceptible to post-SBRT deterioration in liver function [26]. In our cohort, more than half of the patients in both groups were classified as mALBI grade 2a or worse at baseline. Therefore, the lack of statistical significance in the very elderly group may also be attributable to limited statistical power.

The present study has several limitations. First, the sample size was relatively small. The number of variables included in the multivariate Cox model was limited because of the relatively small number of events. Although propensity score matching was performed to adjust for baseline characteristics, complete balance was not achieved for all variables. Nevertheless, most prognostic factors demonstrated standardized mean differences within 0.1 to 0.3, which are generally considered to indicate acceptable balance. However, tumor location and GTV remained slightly imbalanced after matching. Although previous studies have reported tumor size as a potential prognostic factor, the impact of GTV on outcomes remains to be fully elucidated [31]; this imbalance should therefore be considered when interpreting the survival results of the PSM cohort. Second, patient selection by treating physicians may have introduced selection and referral biases, potentially contributing to the numerically favorable OS observed in the very elderly group. In clinical practice, elderly patients with relatively preserved liver function and general condition are more likely to be referred for SBRT, whereas frailer patients may not have been considered candidates for this treatment. Third, the retrospective nature of the study makes it inherently susceptible to selection and indication biases, and some data were missing in a subset of patients. Fourth, a substantial proportion of patients presented with recurrent HCC, and detailed information regarding prior treatment history was limited. This may have influenced outcomes, particularly OS. Fifth, the follow-up period was limited. Therefore, the long-term durability of tumor control and the incidence of late toxicities after SBRT could not be fully evaluated. Further studies with longer follow-up are warranted.

## 5. Conclusions

With the growing proportion of very elderly patients in clinical practice, the need for safe and effective treatment options for HCC is increasing. Our findings suggest that SBRT may represent a feasible alternative treatment option for carefully selected very elderly patients with treatment-naïve or recurrent solitary HCC, with an acceptable safety profile.

## Figures and Tables

**Table 1:** Dose constraints for organ at risk.

Organ	Constraints
Spared liver	V_20Gy_ < 20%
Stomach	V_26.5Gy_ < 5 cm^3^
Duodenum	V_18.5Gy_ < 5 cm^3^ and V_14.5Gy_ < 10 cm^3^

Vx, volume of an organ receiving at least x Gy.

**Table 2:** Patient characteristics in the very elderly group and the control group.

Patient Characteristics	Very Elderly Group (*n* = 41)	Control Group (*n* = 76)	*p*-Value
Age (years)	84.0 [81.0–86.0]	74.0 [69.8–76.0]	**<0.001**
Male, n (%)	22 (53.7)	54 (71.1)	0.070
Performance status, n (%)			**0.040**
0	23 (56.1)	57 (75.0)	
1	18 (43.9)	19 (25.0)	
Etiology of underlying liver disease, n (%)			0.136
HBV	0 (0.0)	9 (11.8)	
HCV	26 (63.4)	40 (52.6)	
ALD	5 (12.2)	6 (7.9)	
MASH	9 (22.0)	19 (25.0)	
Others	1 (2.4)	2 (2.6)	
Comorbidities, n (%)			
Hypertension	30 (73.2)	46 (60.5)	0.224
Hyperlipidemia	9 (22.0)	16 (21.1)	1.000
Diabetes mellitus	16 (39.0)	32 (42.1)	0.845
Location group, n (%)			0.554
S1	5 (12.2)	10 (13.2)	
S2/3	10 (24.4)	15 (19.7)	
S4	4 (9.8)	16 (21.1)	
S5/6	8 (19.5)	10 (13.2)	
S7/8	14 (34.1)	25 (32.9)	
Tumor diameter (mm)	20.0 [13.0–28.0]	16.5 [13.0–25.0]	0.351
Treatment-naïve HCC, n (%)	21 (51.2)	38 (50.0)	1.000
Prior TACE, n (%)	12 (29.3)	21 (27.6)	1.000
Previous treatment modalities for target HCC, n (%)			
RFA	13 (31.7)	22 (28.9)	0.833
TACE	16 (39.0)	35 (46.1)	0.559
Recurrent HCC, n (%)	29 (70.7)	56 (73.7)	0.829
Previous treatment modalities for non-target HCC, n (%)			
Surgery	5 (12.2)	13 (17.1)	0.596
RFA	26 (63.4)	52 (68.4)	0.682
TACE	27 (65.9)	56 (73.7)	0.390
ALBI score	−2.57 [−2.69 to −2.28]	−2.51 [−2.88 to −2.04]	0.964
mALBI grade, n (%)			0.728
1	19 (46.3)	33 (43.4)	
2a	13 (31.7)	19 (25.0)	
2b	9 (22.0)	23 (30.3)	
3	0 (0)	1 (1.3)	
Child–Pugh score, n (%)			0.715
5	33 (80.5)	51 (67.1)	
6	5 (12.2)	14 (18.4)	
≥7	3 (7.3)	11 (14.5)	
ALT (U/L)	16.0 [13.0–25.0]	23.0 [16.8–30.0]	**0.003**
Platelet count (×10^4^/μL)	14.1 [10.5–18.6]	13.0 [8.9–16.1]	0.147
AFP (ng/mL)	6.0 [2.8–14.7]	6.3 [4.1–28.6]	0.370
DCP (mAU/mL)	33.0 [19.0–62.0]	34.0 [23.0–133.5]	0.271
SBRT dose, n (%)			0.104
30 Gy/5 fractions	2 (4.9)	1 (1.3)	
35 Gy/5 fractions	15 (36.6)	16 (21.1)	
40 Gy/5 fractions	24 (58.5)	59 (77.6)	
Gross tumor volume (cm^3^)	4.8 [1.8–13.4]	4.9 [2.6–11.8]	0.751
Planning target volume (cm^3^)	20.9 [11.2–45.2]	20.0 [13.1–31.9]	0.754

HBV, hepatitis B virus; HCV, hepatitis C virus; ALD, alcoholic liver disease; MASH, metabolic dysfunction-associated steatohepatitis; HCC, hepatocellular carcinoma; TACE, transcatheter arterial chemoembolization; RFA, radiofrequency ablation; ALBI score, albumin–bilirubin score; mALBI grade, modified albumin–bilirubin grade; ALT, alanine aminotransferase; AFP, alpha-fetoprotein; DCP, des-γ-carboxy prothrombin; SBRT, stereotactic body radiation therapy. Bold values indicate statistical significance (*p* < 0.05).

**Table 3:** Patient characteristics in the very elderly group and the control group after propensity score matching.

Patient Characteristics	Very Elderly Group (*n* = 30)	Control Group (*n* = 30)	*p*-Value	SMD
Age (years)	84.0 [81.0–85.8]	73.5 [70.0–76.8]	**<0.001**	2.724
Male, n (%)	19 (63.3)	17 (56.7)	0.792	0.136
Performance status, n (%)			0.785	0.142
0	19 (63.3)	21 (70.0)		
1	11 (36.7)	9 (30.0)		
Viral-related liver disease, n (%)	19 (63.3)	22 (73.3)	0.580	0.216
Location group, n (%)			0.224	0.487
S1	5 (16.7)	1 (3.3)		
Left lobe	7 (23.3)	6 (20.0)		
Right lobe	18 (60.0)	23 (76.7)		
Tumor diameter (mm)	17.0 [13.0–25.3]	19.0 [14.0–27.0]	0.790	0.049
Treatment-naïve HCC, n (%)	15 (50.0)	16 (53.3)	1.000	0.067
Prior TACE, n (%)	6 (20.0)	8 (26.7)	0.761	0.158
Previous treatment modalities for target HCC, n (%)				
RFA	10 (33.3)	7 (23.3)	0.567	0.223
TACE	12 (40.0)	13 (43.3)	1.000	0.068
Recurrent HCC, n (%)	23 (76.7)	23 (76.7)	1.000	<0.001
Previous treatment modalities for non-target HCC, n (%)				
Surgery	5 (16.7)	3 (10.0)	0.706	0.197
RFA	20 (66.7)	21 (70.0)	1.000	0.072
TACE	21 (70.0)	22 (73.3)	0.771	0.132
ALBI score	−2.46 [−2.66 to −2.26]	−2.51 [−2.87 to −2.35]	0.337	0.190
mALBI grade, n (%)			0.713	0.247
1	11 (36.7)	12 (40.0)		
2a	11 (36.7)	13 (43.3)		
2b	8 (26.7)	5 (16.7)		
Child–Pugh score, n (%)			0.794	0.188
5	23 (76.7)	25 (83.3)		
6	5 (16.7)	4 (13.3)		
≥7	2 (6.7)	1 (3.3)		
ALT (U/L)	16.0 [13.0–25.8]	18.5 [13.8–25.0]	0.728	0.039
Platelet count (×10^4^/μL)	13.9 [10.6–19.4]	14.7 [10.4–17.1]	0.641	0.165
AFP (ng/mL)	5.9 [2.7–10.2]	5.9 [4.0–22.5]	0.391	0.144
DCP (mAU/mL)	27.0 [18.3–57.0]	30.5 [23.0–99.8]	0.133	0.010
SBRT dose, n (%)			1.000	0.070
35 Gy/5 fractions	10 (33.3)	11 (36.7)		
40 Gy/5 fractions	20 (66.7)	29 (63.3)		
Gross tumor volume (cm^3^)	4.4 [0.41–47.4]	9.7 [0.5–52.9]	0.212	0.332
Planning target volume (cm^3^)	18.5 [4.8–445.3]	25.0 [3.0–107.2]	0.355	0.121

SMD, standardized mean difference; HCC, hepatocellular carcinoma; TACE, transcatheter arterial chemoembolization; RFA, radiofrequency ablation; ALBI score, albumin–bilirubin score; mALBI grade, modified albumin–bilirubin grade; ALT, alanine aminotransferase; AFP, alpha-fetoprotein; DCP, des-γ-carboxy prothrombin; SBRT, stereotactic body radiation therapy.

**Table 4:** Univariate and multivariate Cox regression analysis of factors associated with OS.

	Univariate Analysis			Multivariate Analysis		
Variable	HR	95% CI	*p*-Value	HR	95% CI	*p*-Value
Age (≥80 years)	0.443	0.183–1.075	0.072	0.476	0.185–1.229	0.125
Gender (male)	2.367	1.027–5.456	**0.043**	3.191	1.274–7.988	**0.013**
Performance status	1.094	0.519–2.307	0.814	1.142	0.510–2.553	0.747
Viral-related liver disease	0.518	0.259–1.037	0.063			
Tumor diameter (mm)	1.008	0.974–1.043	0.662	1.038	0.993–1.084	0.097
Treatment-naïve	0.800	0.400–1.601	0.529			
Recurrent HCC	0.554	0.246–1.247	0.154			
Prior TACE	0.569	0.219–1.477	0.247			
ALBI score	2.526	1.235–5.166	**0.011**	2.958	1.441–6.072	**0.003**
Child–Pugh class (B)	1.421	0.546–3.697	0.472			
AFP (ng/mL)	0.999	0.997–1.002	0.541			
DCP ≥ 100	1.767	0.839–3.719	0.134			
Treatment protocols of SBRT (Gy)	1.077	0.927–1.250	0.333			
Gross tumor volume (cm^3^)	1.023	0.995–1.052	0.114			
Planning target volume (cm^3^)	1.008	0.994–1.022	0.258			

OS, overall survival; HR, hazard ratio; CI, confidence interval; HCC, hepatocellular carcinoma; TACE, transcatheter arterial chemoembolization; ALBI score, albumin–bilirubin score; AFP, alpha-fetoprotein; DCP, des-γ-carboxy prothrombin; SBRT, stereotactic body radiation therapy. Bold values indicate statistical significance (*p* < 0.05).

## Data Availability

All data associated with the present study will be available from the corresponding authors upon reasonable request.

## Supplementary Materials

The following supporting information can be downloaded at https://www.mdpi.com/article/10.3390/cancers18111809/s1, Figure S1: Cumulative incidence of local tumor progression accounting for competing risks. No significant difference was observed between the two groups (HR 0.75; 95% CI, 0.20–2.82; *p* = 0.670). Figure S2: Cumulative incidence of distant recurrence accounting for competing risks. No significant difference was observed between the two groups (HR 0.79; 95% CI, 0.50–1.27; *p* = 0.340). HR, hazard ratio; CI, confidence interval. Figure S3: Individual patient trajectories of ALBI scores during follow-up after SBRT. ALBI, albumin-bilirubin.

## Abbreviations

The following abbreviations are used in this manuscript:

AFP	α-fetoprotein
ALBI	albumin–bilirubin
CI	confidence interval
CIRT	carbon-ion radiation therapy
CT	computed tomography
DCP	des-γ-carboxy prothrombin
ECOG	Eastern Cooperative Oncology Group
GTV	gross tumor volume
HCC	hepatocellular carcinoma
HR	hazard ratio
IMRT	intensity-modulated radiation therapy
ITV	internal target volume
IQR	interquartile range
LTP	local tumor progression
MRI	magnetic resonance imaging
MWA	microwave ablation
OS	overall survival
PSM	propensity score matching
RFA	radiofrequency ablation
SBRT	stereotactic body radiation therapy
TACE	transcatheter arterial chemoembolization
