# Supplementary material for: Safety and Efficacy of Stereotactic Body Radiation Therapy in Very Elderly Patients (≥80 Years) with Solitary Hepatocellular Carcinoma
Source: Cancers (Basel). 2026 Jun 1;18(11):1809. doi: 10.3390/cancers18111809 (PMC13255915; doi:10.3390/cancers18111809)
Supplement: Supplementary file 1 [file cancers-18-01809-s001.zip › cancers-4328914-supplementary.pdf]

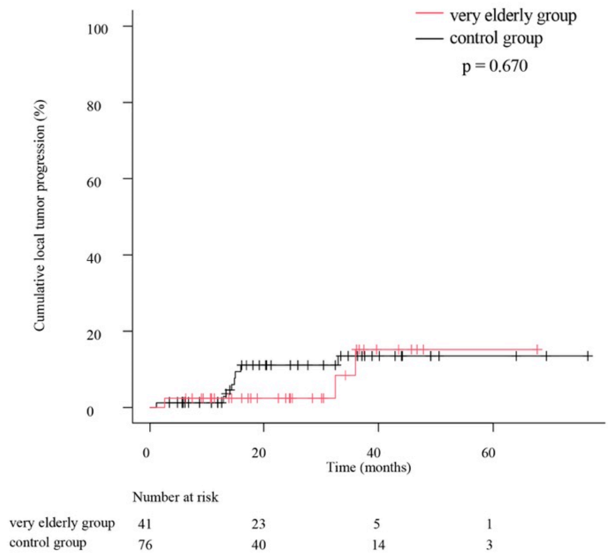

Figure S1.

Cumulative incidence of local tumor progression accounting for competing risks. No significant difference was observed between the two groups (HR 0.75; 95% CI, 0.20–2.82; p = 0.670).

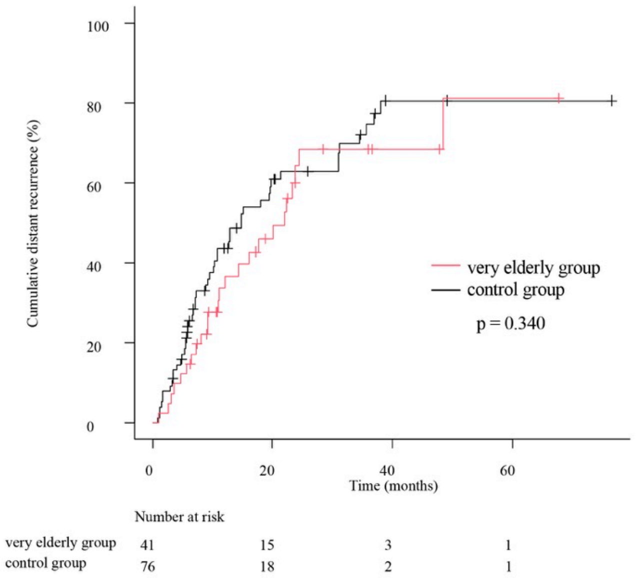

Figure S2.

Cumulative incidence of distant recurrence accounting for competing risks. No sig-nificant difference was observed between the two groups (HR 0.79; 95% CI, 0.50–1.27; p = 0.340). HR, hazard ratio; CI, confidence interval.

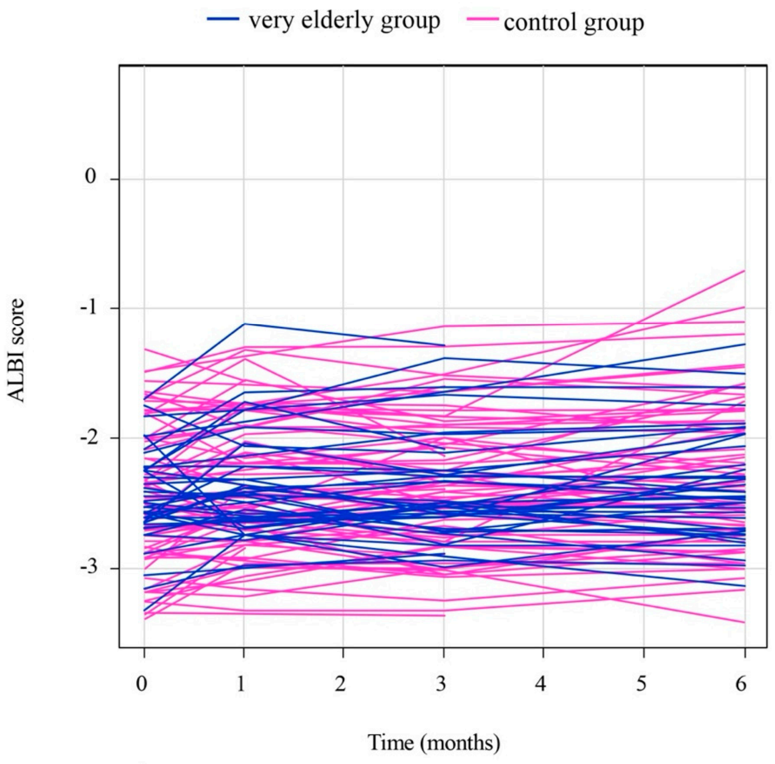

Figure S3.
Individual patient trajectories of ALBI scores during follow-up after SBRT. ALBI, albumin-bilirubin.
